# Familial Sinistrals Avoid Exact Numbers

**DOI:** 10.1371/journal.pone.0059103

**Published:** 2013-03-27

**Authors:** Uli Sauerland, Nicole Gotzner

**Affiliations:** 1 Department of Linguistics, Harvard University, Cambridge, Massachusetts, United States of America; 2 Center for General Linguistics, Berlin, Germany; 3 Department of German Linguistics, Humboldt University, Berlin, Germany; University of Cambridge, United Kingdom

## Abstract

We report data from an internet questionnaire of sixty number trivia. Participants were asked for the number of cups in their house, the number of cities they know and 58 other quantities. We compare the answers of familial sinistrals – individuals who are left-handed themselves or have a left-handed close blood-relative – with those of pure familial dextrals – right-handed individuals who reported only having right-handed close blood-relatives. We show that familial sinistrals use rounder numbers than pure familial dextrals in the survey responses. Round numbers in the decimal system are those that are multiples of powers of 10 or of half or a quarter of a power of 10. Roundness is a gradient concept, e.g. 100 is rounder than 50 or 200. We show that very round number like 100 and 1000 are used with 25% greater likelihood by familial sinistrals than by pure familial dextrals, while pure familial dextrals are more likely to use less round numbers such as 25, 60, and 200. We then use Sigurd’s (1988, *Language in Society*) index of the roundness of a number and report that familial sinistrals’ responses are significantly rounder on average than those of pure familial dextrals. To explain the difference, we propose that the cognitive effort of using exact numbers is greater for the familial sinistral group because their language and number systems tend to be more distributed over both hemispheres of the brain. Our data support the view that exact and approximate quantities are processed by two separate cognitive systems. Specifically, our behavioral data corroborates the view that the evolutionarily older, approximate number system is present in both hemispheres of the brain, while the exact number system tends to be localized in only one hemisphere.

## Introduction

How are numbers mentally processed? Dehaene and others propose a model of amount cognition based on at least two systems: one capable only of representing approximate quantities up to infinity, the other representing a finite set of small exact numbers [1], [2]. [2] suggest that higher integers in adults involve the exact system, but also language and arithmetic. In a similar vein, a review of neuro-imaging evidence finds that distinct brain regions are involved in different aspects of number processing [3] (see also [4]). In particular, a bilateral region, the horizontal segment of the intraparietal sulcus (hIPS) is argued to be the core approximate quantity system. But [3] identify a unilateral region – the left angular gyrus – involved in the verbal processing of numbers.

Language is also well-known to be more likely to be left-lateralized ([5] and much subsequent research). However, the likelihood and degree of the lateralization of language depend on a variety of factors. Relevant for the following are handedness (sinistral vs. dextral) [6] and familial sinistrality, i.e. whether a subject has a left-handed blood relative (FS+ vs. FS–) [7]–[10]. Concerning handedness, [11] report that more than 90% of dextrals and only 70% of sinistrals show left-lateralization of language. Concerning familial influence (i.e. FS+ vs. FS–), [12] show an effect on language lateralization among both sinistrals and dextrals, with FS– increasing the likelihood of left-lateralization in both groups in studies of aphasics. Handedness and familial sinistrality have also been shown to correlate with differences in language behavior (e.g. [13]–[15]). Specifically, [16] argue that, during sentence comprehension, FS+ dextrals access individual words and semantic representations more readily while FS– dextrals first emphasize syntactic representations. [17] shows that FS+ or sinistral individuals generally show lower sensitivity to some grammatical violations.

Following these two lines of research, it is interesting to investigate the relationship of number use and laterality. Like [17], we compare two groups: on the one hand, FS– dextrals and, on the other hand, all others (i.e. FS+ dextrals, FS– sinistrals, and FS+ sinistrals). Compared to the FS– dextrals, the second group is known to have a lower likelihood of left lateralization of language. Furthermore, the two groups both account for about half of the population, which makes it easy to select comparable samples. We use the terms *pure familial dextrals* and *familial sinistrals* to refer to the two groups in the following. We expect that the cognitive effort of using exact numbers increases in familial sinistrals because language and exact numbers may be more distributed over both hemispheres. The cognitive effort of using round numbers, however, should not be affected by familial sinistrality since the approximate quantity system is present in both hemispheres.

The relationship between laterality and cognitive abilities has been controversially discussed from a number of different perspectives [18]. One review of several studies that investigate the relationship of laterality and general cognitive skill concludes that dextrality or sinistrality per se do not cause higher achievement, while extreme lateralization has a small positive effect on general cognitive skill [19]. More specifically for mathematical skill, [20] report a positive correlation of arithmetic ability with sinistrality in 9–11 year old school-children. [21], however, find no general correlation between sinistrality and math skill. In their study, they use the score on an US-American standardized high-school mathematics exam, the SAT-M, as a measure of mathematical skill. They report that in a group of 468 college students, laterality itself is not a significant factor in determining SAT-M scores, but the interaction of laterality and the age of onset of puberty is. Taken together, these results indicate a complex relationship between sinistrality and general mathematical skill where age is an important factor as well, rather than the straightforward correlation Dehaene’s model seems to predict. However, the complexity may be due to the very high level tasks both [20] and [21] use. These may rest on a multitude of underlying skills and furthermore the increased effort of using exact numbers predicted by Dehaene’s model may be compensated by other skills and training over life-time.

In this study, we examine the use of round and exact numbers for approximation. Round numbers are numbers that are more frequently used for approximation. Which numbers are felt to be round depends on the base of the number system. For example, in a language with a decimal base 100 is a round number, but in the Babylonian base 60 system still present in the 60 minutes per hour unit, 15 and 30 are rounder than 25 and 50, which are round in the decimal system. Sigurd [22] suggests the formula in 1 as an index of roundness 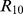 of a number 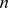 in the decimal system: the greater 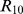 of 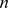 the rounder 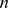 is. Since we are only looking at the decimal systems, we don’t give the general formula of [22] for arbitrary base. Also, [22] doesn’t provide the concise definition in 1, but a mathematically equivalent formulation.
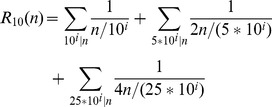
(1)


For numbers smaller than 2500, Sigurd’s index can be computed as in 2, where 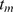 be 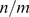 if 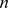 is divisible by 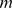, and 0 otherwise. Fractions that lead to division by 0 are to be left out when using the formula in 2. 3 shows how 2 is computed for 500. “Text S1” contains an implementation of this function in the R computer language.
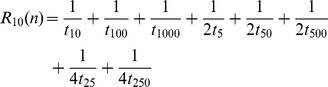
(2)

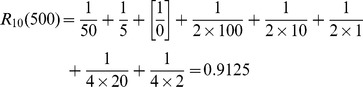
(3)


Sigurd’s formula captures that, in addition to powers of the base (10, 100, 1000, ), also halves and quarters of these powers are basic round numbers if they are whole numbers (5, 50, 500, 25, 250, ), but halves to a lesser degree than the whole powers of the base, and quarters to an even lesser degree. Furthermore, Sigurd assigns higher roundness to small multiples of the basic round numbers, for example 25 (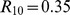) or 200 (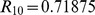), than to greater multiples like 75 (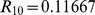) or 800 (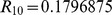). For all positive numbers not divisible by 5, 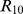 has the value 0. Sigurd shows that the frequency of use of number words and numerical expressions correlates with his roundness index. We use Sigurd’s index in the following to measure the roundness of a number.

## Results

We evaluate the results from 200 participants in an online survey on the Amazon Mechanical Turk crowd-sourcing platform. The survey asked for number trivia like *How many cups do you have in your house?* and *How many students were there in your primary school?*. In addition, we asked some basic demographic information, specifically whether the participants were left-handed or had a close, left-handed blood-relative. Our goal was to investigate the relation between familial laterality and the use of round numbers. The comments of the subjects showed no awareness of the purpose of the investigation. Most reported their participation to have been interesting.

While the questionnaire was presented in English, native speakers of other languages were not excluded since only the decimal base of the number system plays a role for our predictions. The languages used for counting of all participants were decimal languages according to [23]. One subject was a native speaker of Yoruba, which has a base 20 system. However, this subject resided in the United States and used English for counting, and hence is included here as user of a decimal language. The five languages for which more than two native speakers participated are shown in table 1.

**Table 1:** Subjects per Language.

language	# subjects
English	118
Hindi/Urdu	25
Tamil	20
Filipino/Tagalog	5
Malayalam	5
others	32

We only consider estimates in the range between 20 and 1000 for the following analysis because for smaller numbers the subjects may actually have known the precise answer to the question and greater numbers were rare outliers (Only 1.3% of the responses were greater than 1000). This restriction left us with a corpus of 3412 responses of familial sinistrals and 4329 responses of pure familial dextrals.

**Figure 1:**
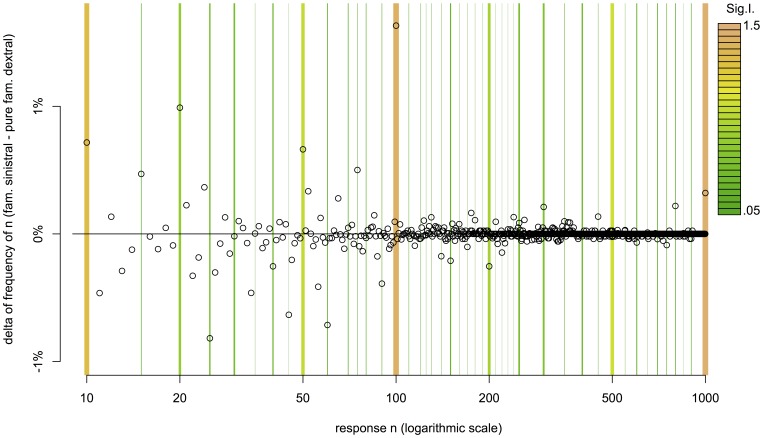
Difference between Familial Sinistrals and Pure Familial Dextrals in Frequency (Percentage-Points) of Numeral 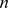 among all Numerals from 10 to 1000. Familial sinistrals responded 100 with 1.6% greater frequency than pure familial dextrals (8.1% vs. 6.4%), 20 with 1.0% (6.7% vs. 5.7%), and 1000 with 0.3% (1.4% vs. 1.1%), but 25 with 0.8% lower frequency (3.3% vs. 4.1%). The vertical bars mark the round numbers, where bar width and color indicate the Sigurd roundness index.

A Welch two-sample t-test shows no significant difference at the 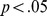 level in mean estimate between the familial sinistrals and pure familial dextrals (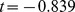, 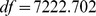, p-value 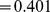, mean estimate of pure familial dextrals 136.24, mean estimate of familial sinistrals 132.86). We find a difference in the use of round numbers between the two laterality groups as shown in Figure 1.

**Figure 2:**
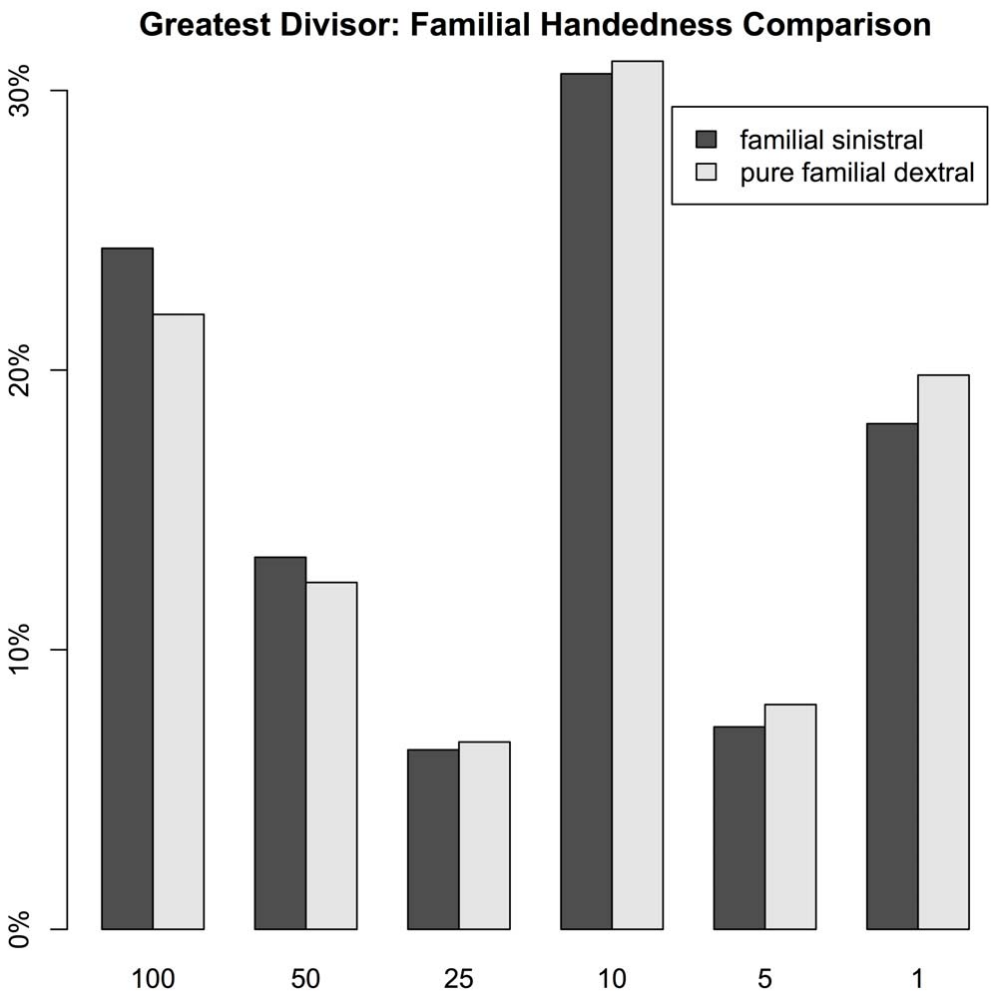
Comparison of Greatest Divisor Frequency by Familial Laterality. A greater percentage of all the answers by familial sinistrals were multiples of 100 or 50 than for pure familial dextrals. Pure familial dextrals more frequently used numbers that were only divisible by 25, 10, 5, or 1, but not by 100 or 50.

We computed two statistics to test for the significance of the effect. On the one hand, we compared the number of responses with greatest divisor 50 or 100 with all other responses. Though this conservative computation doesn’t capture the effect mentioned above that higher multiples of a round base (e.g. 900 and 75) are less round than lower multiples, the comparison shows the difference between familial sinistrals and pure familial dextrals to be significant at the p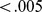 level by the G-test (G(1) = 8.836, p = 0.003). Figure 2 illustrates the comparison of frequency by the greatest divisor from the list of 100, 50, 20, 10, 5, and 1. On the other hand, we compared the Sigurd-scores of the two groups. For the familial sinistral group the average Sigurd score is 0.45, as compared to 0.41 for the pure familial dextral group. A two sample Wilcoxon rank sum test with continuity correction shows this difference to be significant as well with p = 0.0002 (W = 7743933). “Text S1” discusses other demographic factors like gender and shows that these did not affect the roundness of the responses. “Text S1” also presents a third statistical analysis corroborating our main finding.

## Discussion

The initial hypothesis was that exact numbers require a greater cognitive effort for familial sinistral individuals. This hypothesis was based on the assumption that familial sinistrals show more right hemisphere involvement in language related tasks compared to pure familial dextrals. Since [3] suggests the exact number system to rely on a unilaterateral region in the left hemisphere, familial sinistrals were assumed to be less likely to use exact numbers. The results confirm our initial hypothesis: We found that familial sinistral individuals use exact numbers less frequently than pure familial dextral individuals in an estimation task.

Why would familial sinistrals avoid exact numbers in the task more frequently than the right-laterals? We assume that both right- and left-laterals have only approximate knowledge of the number trivia that were elicited by the questionnaire. The reason some subjects chose to respond with an exact number, we assume, is to achieve a rhetorical effect. For example, giving an exact response when it is clear that the subjects cannot have such exact knowledge can indicate amusement or annoyance with the task or that they consider the number unimportant. Overprecision can also indicate that the number is considered to be important as in *‘It’s been 31 days, 3 hours and 12 minutes since I last saw you!’*, however, this seems unlikely to have been the case in our questionnaire. A similar effect of being overly precise is observed in everyday conversation as in the following constructed question-answer pair: *How tall are you?* – *‘I’m exactly 208 cm and 2.68 mm tall.’* Krifka argues that only round numbers are linked with an approximate interpretation [24]. Krifka’s proposal predicts that subjects who respond with an exact number must start with a round number, but then change it by a small quantity to respond with an exact number in the vicinity of the round number. This requires relating the approximate quantity, an exact number, and language. We surmise that establishing this relationship requires a greater cognitive effort by left-lateral individuals because the relevant cognitive functions are more likely to be localized in different hemispheres of the brain.

Another question our results address is the extent to which the numerical concepts related to integers greater than five are dependent on language. As we mentioned, [4] show that the leftward lateralization of counting and linguistic processing in right-handers are correlated in two regions of the cortex and take their results to support the claim that acquiring linguistic symbols affects the cerebral organization of the arithmetic network. This hypothesis is also supported by several studies which demonstrate that the acquisition of linguistic symbols leads to a refinement of the quantity code in the left hIPS [25], [26]. [27] argue that this differential precision in number coding in the two hemispheres might reflect an interaction with an exact verbal code for number in the language-dominant left hemisphere. Crucially, they provide evidence that numerosity estimation depends on a non-verbal mechanism more active in the right hemisphere, while counting activates additional areas in the left hemisphere. The task we used was designed to elicit approximate numerosity estimation. The exact responses participants chose to give are likely to be based on a counting mechanism which requires coordinating non-verbal numerosity estimation abilities and language. Now, following [27], familial sinistrals would appear to be more inclined to use round numbers as their approximate number system might interact with verbal representations to a lower extent. In pure familial dextrals, on the other hand, the left hemisphere is dominant and therefore seems to encourage precise numerical responses.

## Methods

### Ethics Statement

The research reported here was begun while the first author was employed at the Stanford University Linguistics Department in 2009. Ethical approval was given by the Stanford Non-Medical IRB to a Linguistics Department protocol for multiple experiments (protocol # IRB-10833). Written informed consent within this protocol was deemed to be unnecessary for online surveys on Mechanical Turk since it would conflict with the anonymity of participants and participation was voluntary, not expected to cause any harm or discomfort, and could be interrupted by the participants at any point. Participants received information regarding their rights, especially their right to skip questions or withdraw from participation, and about the research purposes of the study.

### Participants

We recruited 200 subjects for an online experiment on the Amazon Mechanical Turk platform. Subjects received 20 US-cents for their participation. Subjects remained anonymous, but we gathered basic demographic information about gender, age, country of origin, education status, native language, and the language used for counting. In addition, we asked the question ‘Are you left-handed or is one of your blood-relatives (father, mother, brothers, sisters, grandparents, aunts, uncles) left-handed?’. Those answering *yes* to this question we refer to as *familial sinistrals*, while those answering *no* we refer to as pure familial dextrals. We did not investigate degree of lateralization because we expected the number of true sinistrals among our subjects to be too small for a meaningful comparison given a prevalence of true sinistrality below 10% [28]. Table 2 shows the basic distribution of subjects according to familial sinistrality. The 4 subjects who did not indicate a familial laterality are excluded from further analysis.

**Table 2:** Overview of participants.

	pure familial dextral	familial sinistral	no response	overall
number of subjects	114	82	4	200
mean age^1^ (SD)	32.25 yrs (10.91)	32.92 yrs (10.05)	25.67 yrs (7.23)	32.42 yrs (10.52)
proportion male	60.56%	53.65%	50.00%	57.50%
prop. missing responses (SD)	2.65% (10.26)	0.83% (2.71)	0.83% (1.67)	1.87% (7.98)
prop. responses 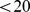 (SD)	32.75% (20.79)	28.62% (17.73)	40% (24.31)	31.20% (19.70)
prop. responses 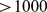 (SD)	1.32% (2.19)	1.20% (2.00)	1.25% (1.60)	1.27% (2.10)
mean work time (SD)	12.58 min (7.24)	11.23 min (4.49)	10.22 min (3.23)	11.98 min (6.22)

1 Subjects were actually asked their year of birth. Age as reported here was computed by subtracting this value from 2009 as testing took place summer/fall 2009. 4 subjects of each laterality group did not enter a year of birth, as did one of the no response group.

In our main analysis, we also excluded responses outside of the 20–1000 range as mentioned above. The portion of responses smaller than 20 was about 4% greater in the pure familial dextral group than in the familial sinistral group, which was significant by the G-test (G(1) = 22.92, p<.00001). The difference is entirely accounted for by the greater occurrence of responses smaller than 10 in the pure familial dextrals: While only 13.1% of all familial sinistrials’ responses were smaller than 10, 18.4% of the pure familial dextrals’ responses were. The difference is in part predicted by our main hypothesis, which predicts familial sinistrals to prefer to respond 10 over exact numerals in the 6 to 9 range. As predicted familial sinistrals respond 10 more frequently than pure familial dextrals (see figure 1). However, this can only in part explain the difference in proportion of responses smaller than 10, and we leave the explanation of this laterality difference to future work. Because we excluded all answers below 20, the main effect we observe is independent of the higher frequency of small number among pure familial dextrals. As we report above, the means of the in-range responses of the two groups do not differ significantly and dextrals actually give slightly greater in-range responses on average. The difference in gender between the two groups is not significant by the G-test (G(1) = 0.92, p-value = 0.34). Our finding contrasts with a significantly greater tendency for sinistrality among males reported in [29]. However, our data only speak to familial sinistrality and [30] report a greater fertility of dextral females. Finally, subjects of the pure familial dextral group spent on average slightly longer to complete the questionnaire, however, the difference between the two groups is not significant at the 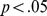 level (Welch Two Sample t-test, t(190.2) = 1.602, p = 0.111).

### Materials and Procedure

The experiment consisted out of 60 questions about number trivia about the subjects’ personal experiences. The questions were designed to elicit approximate responses between 20 and 1000 from the subjects. The following shows four sample questions:

1. How many cups do you have in your house?
2. How many times do you cook in a year?
3. How many cities can you name?
4. How many students were there in your primary school?

To make sure to receive a high number of responses in the 20–1000 range, we conducted the experiment in two stages. After collecting data from 50 subjects, we found that 29 questions elicited responses in the 20–1000 range from less than 50% of subjects. We replaced these 29 questions with new questions to generate more relevant responses. The order of the 60 questions was fixed for the initial 50 and subsequent 150 subjects. A full lists of the questions is provided in “Text S1”. The final question of the questionnaire asked the subjects to comment on the experiment.

### Procedure

The experiment was conducted via the Amazon Mechanical Turk (MTurk) online service as already mentioned above. The experiment was designed in HTML via the Web Interface provided by MTurk. The details of the procedure from the subjects’ perspective are determined by the interface of MTurk on which we experimenters had no direct influence. Participation in the experiment was posted along with other tasks on offer on the main MTurk page for MTurk workers. Such tasks are mainly posted by companies and include image classification, rewriting on product descriptions, transcription of audio recordings, and many others. On this page, the experiment was listed as “How many times a month do you fart? and 59 other fun questions.” The experiment was posted under the company name LanguageLab. At the bottom of the questionnaire a link to a separate web-page was given that explained the broad research goals of the questionnaire. Subjects were instructed not to view the link until completing the questionnaire and could only see the link after scrolling down through most of the experimental items.

## Supporting Information

Text S1 The supplementary discussion presents additional statistics, additional demographic factors, a description of some R language computer code segments used in the analysis, and a list of all survey questions used in the study.
(PDF)
