# Supplementary material for: Familial Sinistrals Avoid Exact Numbers
Source: PLoS One. 2013 Mar 27;8(3):e59103. doi: 10.1371/journal.pone.0059103 (PMC3609743; doi:10.1371/journal.pone.0059103)
Supplement: Text S1 — The supplementary discussion presents additional statistics, additional demographic factors, a description of some R language computer code segments used in the analysis, and a list of all survey questions used in the study. (PDF) [file pone.0059103.s001.pdf]

Familial Sinistrals Avoid Exact Numbers
Supplementary Material

Uli Sauerland[1] & Nicole Gotzner[2]

[1] Harvard University / Center for General Linguistics, Berlin
[2] Humboldt University, Berlin

1 Linear Mixed Model Analysis

In addition to the Wilcoxon-test reported in the results section of the paper, we also computed a mixed model analysis of the Sigurd scores. In order to take account of the fact the we used a random sample of subjects and items, we further fitted a linear mixed model with laterality as fixed effect and subjects and items as random effects. The model revealed a main effect of laterality (t = 2.26, p = 0.025) with a BIC value of 7970. The following table shows the intercept and fixed effect with respective standard errors, t-values and p-values based on Markov Chain Monte Carlo Sampling.

Table 1: **Results of regression analysis**

Fixed Effect	Estimate	Std.Error	t-value	pMCMC
(Intercept)	39.83	2.11	18.86	0.00
Laterality:left	5.85	2.81	2.08	0.025

2 Gender and other factors

Most factors measured such as subject's education level and age did not exert a significant effect on the roundness of estimates. In addition to laterality, the frequency of precise responses was significantly affected by gender as shown by figure 1. This effect, however, can be explained by a bias in the questions to yield higher responses from females. Thus the mean estimate of females was

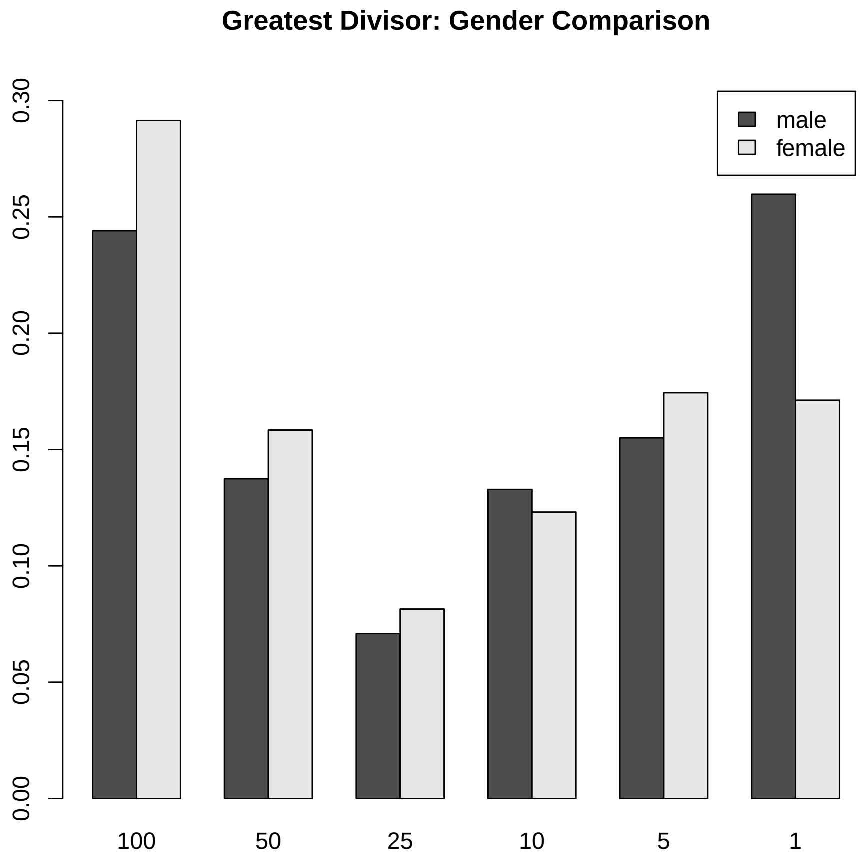

Figure 1: Gender Comparison of Greatest Divisors

182.9 vs. 171.9 for males. We expect that subjects would be more likely to use a round number for a higher value. We therefore computed a linear regression analysis mapping median estimate and mean Sigurd score of male and female subjects. Figure 2 shows the data that the analysis is based on and the regression lines for male and female subjects. The statistical analysis shows an expected significant positive correlation of median estimate and the average Sigurd score of a subject (t = 3.56, p = 0.0005). However, no significant influence of gender is found (t = -1.58, p = 0.116).

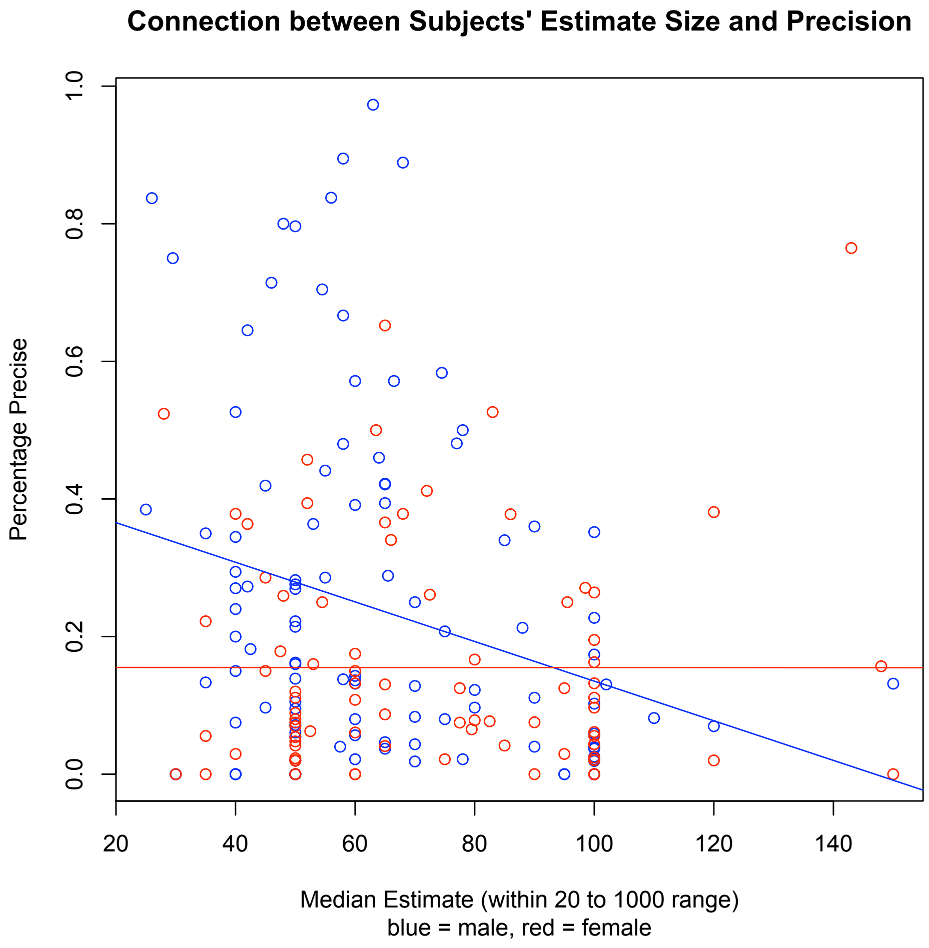

Figure 2: Effects of Median Estimate and Gender on Sigurd Index

3 Code Segments

The following code was used with the R software package. The function *greatest.divisor* returns for a vector of numbers v a vector that counts for how many of the values in v are between 20 and 1000 and have 100, 50, 25, 10, 5, and 1 as their greatest divisor from this list of divisors.

```
greatest.divisor = function(v) { app=table(v);
ac = as.numeric(names(app));
divs= c(sum(app[ac>=20 & ac<= 1000 & ac %% 100 == 0]),
sum(app[ac>=20 & ac<= 1000 & ac %%100!=0 & ac%%50==0]),
sum(app[ac>=20 & ac<= 1000 & ac %% 100 != 0 & ac%%50!=0& ac %% 25 == 0]),
sum(app[ac>=20 & ac<= 1000 & ac%% 50!=0& ac %% 50 != 0 & ac %% 10 == 0]),
sum(app[ac>=20 & ac<= 1000 & ac %% 10 != 0 & ac%%25 !=0& ac %% 5 == 0]),
sum(app[ac>=20 & ac<= 1000 & ac %% 5 != 0]));
names(divs)<-c(100,50,25,10,5,1);
return(divs) }
```

Example Usage:

```
> greatest.divisor(c(200,32,5))
100  50  25  10   5   1
  1   0   0   0   0   1
```

The function *sigurd* returns the Sigurd-Score of a number between 20 and 1000.

```
s1 = function (n,d,w) {if (n %% d == 0) return (d/n/w) else  return(0)}
sigurd = function (n)
  { if (is.na(n)) {return(NA)}
    else
    {return (s1(n,10,1)+s1(n,100,1)+s1(n,1000,1)+s1(n,5,2)+s1(n,50,2)
       +s1(n,500,2)+s1(n,25,4)+s1(n,250,4))} }
```

Example Usage:

```
> sigurd(989)
[1] 0
> sigurd(900)
[1] 0.1597222
> sigurd(800)
[1] 0.1796875
```

```
> sigurd(90)
[1] 0.1388889
```

4 All Questions and Averages

The following table shows that number of responses in the 20–1000 range and the average of the responses in that range. Items were presented to either 50, 150 or 200 subjects as described above. At least one item, *How many times have you ever gotten a hear cut?* contained a typo, but this seems to not have caused a problem for most of the subjects. Only one subject noted the typo in the comments field.

Question	#responses in range	average
How many animal species can you identify?	130	113.8
How many bands or musical groups can you name?	95	116.1
How many bank transactions do you have per year?	158	198.6
How many banknotes have you touched this month?	80	143.4
How many bird species can you identify?	13	47.3
How many books did you read last year?	16	52.9
How many books do you own?	155	146.3
How many buttons are there in your wardrobe?	102	155.4
How many cats did you see last week?	3	82
How many CDs do you own?	150	123.3
How many celebrities do you respect?	10	60
How many chairs, couches and stools do you have in your house?	9	25.7
How many cities can you name?	138	192.2
How many coins in total do you currently have?	94	219.4
How many countries can you name?	120	80.2
How many countries have you visited?	0	0
How many cups of coffee or tea do you drink per month?	135	63.3
How many cups or glasses do you have in your house?	139	51.1
How many days do you work per year?	172	243.8
How many days with rain did you have last year?	170	82.1
How many days with sunshine did you have last year?	180	218.1
How many dogs did you see last week?	8	48.6
How many eggs did you eat last year?	154	138.1

How many e-mails do you receive in an average month?	128	298
How many friends do you have?	21	78.5
How many glasses of beer or wine do you drink per month?	8	65.9
How many houses are in your neighborhood?	127	122.5
How many houses are on your street?	101	76
How many insect species can you identify?	13	116.5
How many keys have you owned in your life?	88	51.7
How many knives are in your house?	13	30.7
How many light bulbs are there in your home?	67	38.8
How many living relatives do you have?	147	96.9
How many loaves of bread do you buy per year?	151	97
How many mammal species can you identify?	19	81.1
How many married people do you know?	110	111.6
How many meals do you eat per month?	187	80.5
How many minutes does it take to the nearest public airport from your house?	159	67.6
How many movie actors can you recognize?	125	85
How many musical instruments can you identify?	18	37.7
How many night have you ever that you didn't sleep at all?	17	53.1
How many nights did you sleep badly last year?	127	104
How many pairs of shoes have you owned in your life?	116	132.2
How many pants or skirts do you own?	16	37.5
How many pens do you have in your house?	21	52.9
How many people are you angry at?	12	174
How many people did you talk with last week?	112	57.7
How many peoples birthdays do you remember?	7	29
How many phone calls did you make last month?	158	120.7
How many phone numbers have you stored somewhere?	165	124.3
How many pieces of fruit did you eat last year?	131	268
How many plant species can you identify?	16	57.3
How many plates or bowls do you have in your house?	145	47.7
How many politicians can you identify by their looks?	112	52.4
How many relatives of yours have you ever met?	158	87.9
How many restaurants have you ever eaten at?	110	233.4
How many shirts do you own?	128	54.7
How many short friends do you have?	11	55.8
How many socks do you own?	18	46.3

How many spoons do you have in your house?	119	44.2
How many students were there in your primary school?	137	354.6
How many tall friends do you have?	1	89
How many teachers did you have?	129	61.7
How many times did you ever get something you really wanted?	11	65.5
How many times did you ever have a headache?	93	272.6
How many times do you burp per month?	21	69.5
How many times do you cook in a year?	145	303.7
How many times do you do laundry in a year?	157	128.5
How many times do you do shopping in a year?	120	113.6
How many times do you fart per month?	142	96.8
How many times do you have guests in a year?	15	95.3
How many times have you been seriously sick?	2	294.5
How many times have you ever been to a doctor?	155	96.3
How many times have you ever been to the dentist?	19	66.9
How many times have you ever burned yourself?	10	40.6
How many times have you ever gotten a hear cut?	111	194.7
How many times have you ever had a fever?	112	77.6
How many times have you taken medication?	98	277.5
How many times have you turned on a computer last year?	125	387.7
How many times in an average week do you look at a clock or watch?	130	160.7
How many times in an average week do you look in the mirror?	86	83.5
How many times last month did you turn on a faucet or tap?	130	162.6
How many times last week did you open a door?	125	107
How many times last week did you turn on a light?	115	61.7
How many trees are there in your neighborhood?	97	156.3
How many vacations lasting a week or longer have you ever taken?	6	37.7
How many vegetarian meals did you eat last month?	22	78
How many web pages have you visited today?	47	77.8
How many windows were there in the building where you went to primary school?	109	119.2

5 Data and Files

The cleaned data sets are changed from the raw data in the following respects:

- We unified capitalization and spelling of languages.
- One respondent entered '100s' once. We changed this to the numerical 100.
- One person entered 10000000000, which we replaced with 10000.
- In some cases -.1 was entered for missing values.

The following data files are included with the appendix:

- `Batch_79873_result_CLEANED.csv`: Cleaned data of Subjects 1 to 50.
- `Batch_81617_result_CLEANED.csv`: Cleaned data of Subjects 51 to 100.
- `Batch_107461_result_CLEANED.csv`: Cleaned data of Subjects 101 to 200.
- `questions.csv`: List of questions for Subjects 1 to 50.
- `questions2.csv`: List of questions for Subject 51 to 100
- `questions3.csv`: List of questions for Subject 101 to 200
- `Survey-version01.html`: Survey html code for subjects 1 to 50.
- `Survey-version02.html`: Survey html code for subjects 51 to 200.
